# Prevalence of Acute Bacterial Infections and Their Antibiotic Sensitivity Pattern in Children With Severe Acute Malnutrition From a Tertiary Care Hospital of Odisha

**DOI:** 10.7759/cureus.65280

**Published:** 2024-07-24

**Authors:** Sanjay Kumar Sahu, Deepti D Pradhan, Rama K Gudu, Suresh K Tripathy, Pravati Jena

**Affiliations:** 1 Pediatrics, Kalinga Institute of Medical Sciences, Bhubaneswar, IND; 2 Neonatology, Institute of Medical Sciences and SUM Hospital, Bhubaneswar, IND

**Keywords:** bacteremia, mid upper arm circumference (muac), urinary tract infection, immunocompromised, severe acute malnutrition (sam)

## Abstract

Background and objective

Malnutrition remains a significant cause of childhood morbidity and mortality worldwide. Severe acute malnutrition (SAM) profoundly affects immune development, physiological functions, and metabolic processes, increasing susceptibility to infections. This study aimed to investigate the prevalence of acute bacterial infections and their antibiotic sensitivity patterns among SAM children admitted to a tertiary care hospital.

Methodology

This prospective observational study was conducted at the pediatric department of Kalinga Institute of Medical Sciences (KIMS), Bhubaneswar, Odisha, from November 2020 to October 2023. The study included 95 children aged 6-59 months meeting WHO criteria for SAM. Participants underwent comprehensive demographic assessments, clinical evaluations, and relevant laboratory tests, including blood and urine cultures with sensitivity testing.

Results

The study found that 82.1% of children had weight-for-height below -3 standard deviations, and 84.21% had mid-upper arm circumference below 115 mm, confirming SAM diagnosis. The most prevalent infections were acute gastroenteritis (47.3%), respiratory tract infections (46.3%), bacteremia (27.4%), and urinary tract infections (26.3%). Positive urine cultures were observed in 25 cases (26.3%), predominantly among females (68%). *Escherichia coli* (40%) and *Klebsiella pneumoniae *(24%) were the most common organisms isolated from urine, with high sensitivity to gentamicin (76%) and meropenem (72%). Blood cultures were positive in 26 cases (27.36%), with *Staphylococcus aureus* ​​​​​​(30.76%) and *Klebsiella pneumoniae *(23%) being predominant. Blood isolates showed significant sensitivity to vancomycin (73%), meropenem (69.2%), and linezolid (65.3%).

Conclusion

Acute gastroenteritis, respiratory tract infections, bacteremia, and urinary tract infections are prevalent among SAM children. *Staphylococcus aureus* was frequently isolated from blood cultures, while *Escherichia coli* were predominant in urine cultures. High sensitivity of urinary isolates to gentamicin and meropenem, and of blood isolates to vancomycin, meropenem, and linezolid, highlights effective antibiotic choices. These findings emphasize the importance of tailored antimicrobial therapy based on local sensitivity patterns to improve clinical outcomes in SAM children.

## Introduction

Malnutrition is a significant contributor to morbidity and mortality among the pediatric population worldwide, leading to an increased risk of death or severe impairment in growth and psychological development. Severe acute malnutrition (SAM) represents an extreme type of malnutrition, resulting in profound physiological and metabolic changes. In response to the scarcity of nutrients and energy, a malnourished child’s metabolism reduces activity and slows down to survive on limited intake, preserving essential body functions. These adaptations affect every cell, tissue, and system. Initially, these reductions do not hinder the body's ability to respond to minor changes but eventually impair its capacity to handle stressful situations like infections.

SAM impairs normal immune system development, making children vulnerable to infections, particularly by opportunistic organisms [1]. The gastrointestinal mucosal integrity and architecture are altered including flattened hypotrophic microvilli, reduced lymphocyte counts in Peyer’s patches, and decreased secretion of immunoglobulin A (IgA) leading to decreased immune defense. This state is named as nutritionally acquired immunodeficiency syndrome [2]. Infections stimulate the immune system, leading to an increased need for anabolic energy and substrates. This creates a detrimental cycle where nutritional status deteriorates, and vulnerability to infections rises.

Malnourished children predominantly experience bacterial infections in the gut, respiratory, and urogenital tracts due to compromised mucosal epithelial integrity. The heightened susceptibility to infections may be the result of a breach of anatomical barriers, reduced cell-mediated immunity, diminished opsonic activity, impaired phagocytosis, and vitamin A deficiency. Therefore, the routine use of antibiotics in the initial management of SAM patients, as recommended by the World Health Organization (WHO), is justified. However, locally prevalent pathogens and their antibiotic susceptibility patterns should be considered before the selection of antibiotics.

Due to the scarcity of recent literature regarding the prevalence of acute bacterial infections and their antibiotic sensitivity pattern in children aged 6 to 59 months with SAM from our region, this study was conducted.

## Materials and methods

This prospective observational study was conducted on patients with SAM admitted to the pediatric department of Kalinga Institute of Medical Sciences (KIMS), Bhubaneswar, Odisha, from November 2020 to October 2023 after obtaining ethical clearance from the institutional ethical committee and informed and written consent from the parents of children. The sample size was calculated using 16% prevalence from a previous study by Abdulsalam et al. with a 7% margin of error and a 5% level of significance, which came out to be 106 patients, so the total sample size taken was 118 [3]. The primary objective was to determine the prevalence of acute bacterial infections and their antibiotic sensitivity pattern in children aged 6 to 59 months with SAM. The secondary objective was to develop an empirical antibiotic regimen for our region based on the observed sensitivity patterns.

Children were enrolled in the study according to the anthropometric measurements, nutritional status, and inclusion criteria. Inclusion criteria were children aged six months to 59 months who were admitted to the pediatric department of KIMS and met the WHO criteria for SAM. The WHO criteria for diagnosis of SAM include weight-for-height/length below -3 standard deviations (SDs) of the median (using the WHO Growth Charts), visible severe wasting, bipedal edema of nutritional origin, or mid-upper arm circumference (MUAC) less than 115 mm [4].

Infants below six months, children above 60 months, and those with secondary malnutrition due to chronic medical or surgical conditions were excluded from the study.

Each participant was evaluated using a predefined proforma that included demographic details and relevant clinical history. A thorough clinical examination was performed, with emphasis on anthropometric measurements (height, weight, and MUAC). Hematological investigations included a complete blood count, C-reactive protein, erythrocyte sedimentation rate (ESR), slide for malaria parasites, liver and kidney function tests, blood culture, and HIV testing. Additional tests included chest X-ray, routine microscopy, and culture and sensitivity tests of urine, stool, and pus.

Urine samples were collected via mid-stream clean catch for children above two years and suprapubic aspiration for children below two years. Blood culture samples were collected at admission, prior to antibiotic administration, using BacT/Alert PF (Pediatric) bottles. Cerebrospinal fluid was examined only in clinically suspected cases of CNS infection or those showing signs of meningeal irritation, including tests for sugar, protein, cytology, gram staining, and culture and sensitivity.

Collected data were recorded in a Microsoft Excel 2007 spreadsheet and analyzed using IBM SPSS Statistics for Windows, Version 21 (Released 2012; IBM Corp., Armonk, New York, United States). Categorical variables were represented by numbers and percentages (%), while quantitative data were presented as means ± SD and as median with interquartile range (25th-75th percentile). The association between quantitative variables was assessed using independent t-tests, and the association between categorical variables was evaluated using chi-square tests. 

## Results

This observational study explores the clinical profile, infectious associations, and antibiotic sensitivity pattern among children diagnosed with SAM. In this study out of 118 children in the age group of 6 months to 59 months, 95 were enrolled and 23 were excluded as per the exclusion criteria. In our study, most of the children i.e. 36 (38%) were in the age group between 6 to 12 months followed by 30 (31.5%) in the 13 to 24 months age group and 29 (30.5%) in the 25 to 59 months age group. The mean value of age (months) of study subjects was 19.43 ± 12.75 with a median (25th-75th percentile) of 15 (9-27). The study population had 52 males (54.73%) and 43 females (45.3%). A total of 78 (82.1%) patients out of 95 had weight for height less than -3 SD. MUAC less than 115 mm was seen in 80 (84.21%) patients. Edema was present in only 14 (14.7%) patients and visible severe wasting was seen in 55 (57.89%) patients. Apart from poor weight gain, the presenting complaints were fever in 68 patients (71.5%), followed by vomiting in 49 patients (51.57%), loose motion in 45 patients (47.3%), cough in 45 patients (47.3 %), and loss of appetite or weight loss in 30 patients (31.57%). Other common presenting symptoms were abdominal distension in 13 patients (13.6%), rash in four patients (4.2%), convulsion in three patients (3.1%), ear discharge in two patients (2.1%), and bleeding in two patients (2.1%). Infections associated with SAM were acute gastroenteritis in 45/95 (47.3%), followed by acute respiratory tract infection in 44/95 (46.3%), bacteremia in 26/95 (27.4%) urinary tract infection (UTI) in 25/95 (26.3%), and malaria in 4/95 (4.2%) patients. Other associated infections were tuberculosis in 3/95 (3.1%), measles in 3/95 (3.1%), meningitis in 2/95 (2.1%) otitis media in 2/95 (2.1%), and HIV in 1/95 (1.05%). Some children presented with skin infections like pyoderma 10/95 (10.5%), scabies 3/95 (3.1%), and candidiasis 2/95 (2.1%). Table 1 depicts the demographic profile and presenting features of children with SAM.

**Table 1:** Demographic profile and presenting features of children with SAM SAM: Severe acute malnutrition

Parameters	n (%)
Demographic profile	
Age 6 to 12 months	36(38%)
Age 13 to 24 months	30(31.5%)
Age 25 to 59 months	29(30.5%)
Male	52(54.7%)
Female	43(45.3%)
Presenting features	
Fever	68(71.5%)
Cough	45(47.3 %)
Vomiting	49(51.57%)
Loose motion	45(47.3%)
Loss of appetite or weight loss	30(31.57%)
Edema	14(14.7%)
Abdominal distension	13(13.6%)
Rash	4(4.2%)
Convulsion	3(3.1%)
Ear discharge	2(2.1%)
Bleeding	2(2.1%)
Pattern of infectious disease	
Acute gastroenteritis	45(47.3%)
Acute respiratory tract infections	44(46.3%)
Bacteremia	26 (27.4%)
Urinary tract infection	25 (26.3%)
Malaria	4(4.2%)
Culture positive	
Blood	26(27.36%)
Urine	25(26.31%)

The total number of urine culture-positive samples out of 95 was 25 (26.3%). Out of these culture-positive urine samples, 17/25 (68%) were of female and 8/25 (32%) were of male.

The commonest organisms isolated from urine culture were *Escherichia coli* 10/25(40%), followed by *Klebsiella pneumoniae* 6/25(24%), *Acinetobacter baumannii* 3/25(12%), *Staphylococcus epidermidis* 2/25(8%), *Staphylococcus aureus* 2/25(8%), *Enterococcus faecalis* 1/25(4%), and *Pseudomonas aeruginosa* 1/25(4%). The organisms isolated from urine were highly sensitive to gentamicin (76%) and meropenem (72%). The sensitivity pattern of other antibiotics was piperacillin+tazobactum (52%), amikacin (48%), and ceftriaxone (40%). Table 2 depicts the organisms isolated from urine and their sensitivity pattern.

**Table 2:** Organisms isolated from urine and their sensitivity pattern

Organisms(n) Drug	Escherichia coli (10)	Klebsiella pneumoniae (6)	Acinetobacter baumannii (3)	Pseudomonas aeruginosa (1)	Staphylococcus aureus (2)	Enterococcus faecalis (1)	Staphylococcus epidermidis (2)	Total 25 n/25(%)
Piperacillin + Tazobactum	5(50%)	4(67%)	2(67%)	1(100%)	1(50%)	0	0	13 (52%)
Ceftriaxone	4(40%)	3(50%)	0	0	1(50%)	1 (100%)	1(50%)	10(40%)
Amikacin	7(70%)	4(67%)	0	1(100%)	0	0	0	12(48%)
Gentamicin	8(80%)	5(83%)	2(67%)	1(100%)	1(50%)	1 (100%)	1(50%)	19(76%)
Ofloxacin	6(60%)	3(50%)	0	0	0	0	0	9(36%)
Amoxyclav	5(50%)	1(17%)	0	0	1(50%)	1 (100%)	1(50%)	9(36%)
Meropenem	8(80%)	4(67%)	2(67%)	1(100%)	1(50%)	1 (100%)	1(50%)	18(72%)
Vancomycin	0	0	0	0	2(100%)	1 (100%)	2(100%)	5(20%)
Linezolid	0	0	0	0	2(100%)	1 (100%)	2(100%)	5(20%)

Out of 95 blood specimens, 26 were culture-positive. The commonest bacterial growth was *Staphylococcus aureus* 8/26 (30.76%), followed by* Escherichia coli *6/26 (23%), and coagulase negative staphylococcus 5/26 (19.23%) all being *Staphylococcus epidermidis, Escherichia coli *4/2(15.38%), and Group D streptococcus (*Enterococcus faecalis*) 3/26 (11.5%).

**Figure 1:**
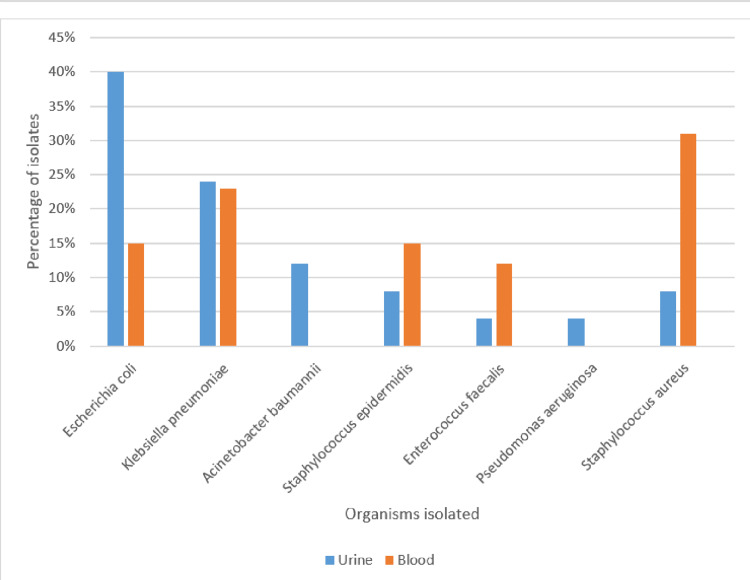
Organisms isolated from urine and blood

The bacteria isolated from the blood culture were sensitive to vancomycin (73%), meropenem (69.2%), and linezolid (65.3%). The other sensitive antibiotics were cotrimoxazole (57.6%), ciprofloxacin (42.3%), and gentamicin (42.3%). Table 3 depicts organisms isolated from blood and their sensitivity pattern. Figure 1 represents the organisms isolated from urine and blood.

**Table 3:** Organisms isolated from blood and their sensitivity pattern

Bacteria (n) Drug	Staphylococcus aureus (8)	Staphylococcus epidermidis (5)	Enterococcus faecalis (3)	Escherichia coli (4)	Klebsiella pneumoniae (6)	Total (26) n/26(%)
Ampicillin	2(25%)	2(20%)	1(33.3%)	1(25%)	0	6/26(23%)
Amikacin	0	0	2(66.6%)	2(50%)	2(33.3%)	6/26(23%)
Ceftriaxone	3(37.5%)	2(40%)	1(33.3%)	3(75%)	1(16.6%)	10/26(38.4%)
Gentamicin	4(50%)	2(40%)	2(66.6%)	2(50%)	1(16.6%)	11/26(42.3%)
Meropenem	6(75%)	3(60%)	2(66.6%)	3(75%)	4(66.6%)	18/26(69.2%)
Vancomycin	8(100%)	5(100%)	2(66.6%)	0	4(66.6%)	19/26(73%)
Linezolid	8(100%)	4(80%)	3(100%)	0	2(33.3%)	17/26(65.3%)
Ciprofloxacin	4(50%)	1(20%)	1(33.3%)	1(25%)	4(66.6%)	11/26(42.3%)
Cotrimoxazole	6(75%)	4(80%)	2(66.6%)	2(50%)	1(16.6%)	15/26(57.6%)

## Discussion

This observational study included 95 children, aged 6 months to 59 months, out of a total of 118 initially considered. The largest age group affected, 6 to 12 months (38%), may be associated with factors such as premature weaning from breastfeeding, inappropriate introduction of complementary feeding, and micronutrient deficiencies [5]. The mean age of the children was 19.43 ± 12.75 months, with a median age of 15 months (interquartile range: 9-27 months). The sex distribution was fairly well-adjusted, with 52 males (54.73%) and 43 females (45.3%).

Besides poor weight gain, the most common presenting complaint was fever in our study [6-8]. However, some studies have found respiratory symptoms to be more common [9]. This may be due to decreased immunity masking overt signs and symptoms leading to varied manifestations.

The study emphasizes the high burden of infections among children with SAM. The most common infections were acute gastroenteritis, acute respiratory tract infections, bacteremia, and UTIs.

Gastroenteritis was the most common infection encountered in SAM children due to the overgrowth of bacteria in their small intestines. They usually present with watery diarrhea and may also have some degree of dehydration [10-12]. Moreover, dehydration directly modifies the anthropometric indices, such as weight, weight-for-height/length, and MUAC. Managing dehydration is critical for reducing morbidity and mortality. Respiratory tract infections are very common in children with SAM as depicted in many studies [13]. The typical presentation of pneumonia may be obscured in malnourished children with minimal respiratory distress, resulting in under-diagnosis and suboptimal treatment [13]. Acute respiratory infections reciprocally worsen the nutritional condition through augmented catabolism, loss of appetite, and nutrient depletion, leading to a vicious cycle of infections and malnutrition [14]. Other respiratory infections commonly encountered in SAM include tuberculosis and measles. Measles is more frequently associated with vitamin A deficiency. 

The prevalence of UTIs in this study is 26% which is similar to the study done by Tiwari et al. (23.37%) [15]. According to global data, there is substantial evidence indicating that children with SAM are at a significantly higher risk of UTI with prevalence rates varying widely from 6% to 37% [16]. UTIs are significantly more common among malnourished children compared to their healthy counterparts, and the likelihood of UTIs increases with the severity of malnutrition [16]. UTIs were noted in one-fourth of the children, with a higher prevalence in females compared to males. It is noteworthy that females constituted a higher percentage of culture-positive urine samples compared to males. This observation could be due to anatomical differences, or varying susceptibility to urinary tract infections between genders. UTIs may lead to acute renal infection and may contribute to systemic sepsis. Repeated UTIs may lead to renal scarring, leading to renal damage. Classical clinical manifestations of UTI may not be apparent in children with SAM and in some cases, only poor weight gain may be the only symptom.

The high prevalence of *Escherichia coli *aligns with its well-established role as a common cause of UTIs in this study [15]. *Klebsiella pneumonia, Acinetobacter baumannii, Pseudomonas aeruginosa,* and *Staphylococcus epidermidis *were also identified but in lower frequencies. *Escherichia coli *and *Klebsiella pneumoniae* are the major contributors to UTIs in SAM children which aligns with reports from previous studies [15,17,18].

In this study, 95 blood specimens were analyzed, and 26 (27.4%) were found to be culture-positive. However, this culture positivity rate is not consistent with findings of a similar study done in Niger, which reports to be 9.1% [19]. The higher yield from the blood culture may be because the children had not received any antibiotics prior to hospitalization.

The analysis of the culture-positive specimens in our study revealed that* Staphylococcus aureus* was the most commonly isolated bacterium, accounting for one-third of the positive cultures. However, non-typhoid Salmonella was identified in over half (57.8%) of cases in a Nigerian study [19]. Other similar studies also support that bacteremia in children with SAM is primarily due to enteric pathogens like Salmonella species and *Escherichia coli *[20-22]. Additionally, a notable portion of these infections is caused by Gram-positive organisms, such as *Streptococci *and* Staphylococcus aureus* [20-22]. However, a meta-analysis of 11 studies, covering community-acquired pediatric bloodstream infections in Africa between 1992 and 2010, found that the most common pathogen was* Staphylococcus aureus *(17.8%) [23]. So the causative agents of bacteremia may vary geographically.

Following *Staphylococcus aureus, Klebsiella pneumoniae *was identified in 23% (6/26) of the positive cultures. It is a significant nosocomial pathogen and is known for its ability to acquire resistance to multiple antibiotics, making infections difficult to treat [24]. The relatively high prevalence of *Klebsiella pneumoniae *in this study indicates a potential concern for hospital-acquired infections, particularly in settings with vulnerable patient populations. *Escherichia coli *was identified in 15.38% (4/26) of the positive cultures, which is consistent with its role as a common cause of bloodstream infections, particularly in community-acquired cases [24]. It is often associated with urinary tract infections, and its presence in the bloodstream can indicate a secondary spread from a primary infection site.

A number of studies support coagulase-negative Staphylococci (CoNS), for example, *Staphylococcus epidermidis* as pathogens [25-28]. CoNS are the normal inhabitants of the skin microbial flora. Any loss in the integrity of normal skin defense barrier, which is commonly seen in SAM, facilitates the entry of these organisms into the bloodstream with resultant bacteremia. CoNS are widely recognized as a major cause of sepsis in critically ill and immunocompromised children. In our study, CoNS accounted for 19.23% (5/26) of the positive cultures. The presence of CoNS as a significant proportion of the isolates suggests the importance of differentiating between contamination and true infection to guide appropriate treatment strategies.

Group D Streptococcus, which includes *Enterococcus faecalis*, was found in 11.5% (3/26) of the positive blood cultures; however, a study done by Singh et al. found it to be 25% [29]. Group D Streptococcus is known to cause endocarditis, UTIs, and intra-abdominal infections. The identification of these organisms highlights the diverse etiology of bloodstream infections and the need for comprehensive diagnostic approaches.

As per a meta-analysis, most guidelines recommend amoxicillin as the first-line therapy in uncomplicated SAM and for complicated SAM there is wide variation in the first-line therapy recommended, including ampicillin/amoxicillin, gentamicin, third-generation cephalosporins, ciprofloxacin, co-amoxiclav, metronidazole and amikacin [30]. Over a period of time, the sensitivity pattern of the antibiotics has changed. Increased misuse of antibiotics results in resistance to commonly used antibiotics. The bacterial isolates in this study show varied sensitivity to the commonly used broad-spectrum antibiotics. *Escherichia coli *isolated from urine was highly sensitive to gentamicin, meropenem, and amikacin. Most of the organisms isolated from urine were highly sensitive to gentamicin and meropenem. *Staphylococcus aureus* isolated from blood was sensitive to vancomycin, linezolid, and meropenem. So meropenem along with vancomycin can be used initially to treat sick children with SAM while awaiting the culture results. However, this cannot be generalized as microbial resistance profiles vary widely in different studies [19,20]. The effectiveness of these antibiotics underscores their role in managing severe infections in malnourished children, though ongoing surveillance for antibiotic resistance remains critical.

Limitations of the study

The small sample size and study done at a single center are the major limitations of this study; hence, the results cannot be generalized.

## Conclusions

This observational study emphasizes the significant burden of infections among children suffering from SAM. The findings highlight a diverse spectrum of infections, including gastroenteritis, respiratory tract infections, UTIs, and bacteremia, with pathogens such as Escherichia coli, Staphylococcus aureus, and Klebsiella pneumoniae prominently identified. Most of the organisms isolated from urine were highly sensitive to Gentamicin and Meropenem and from blood were sensitive to vancomycin, meropenem, and linezolid. Effective management of these infections remains crucial, with recommendations suggesting tailored antibiotic therapy based on local sensitivity patterns, while emphasizing the need for ongoing surveillance to address emerging antibiotic resistance. However, the significant burden of infections and malnutrition highlights the need for comprehensive strategies, including improved nutritional support, timely diagnosis, and effective treatment of infections, to improve outcomes in this vulnerable population.
